# Tetrahydro-iso-alpha Acids Antagonize Estrogen Receptor Alpha Activity in MCF-7 Breast Cancer Cells

**DOI:** 10.1155/2016/9747863

**Published:** 2016-04-12

**Authors:** Maëlle Lempereur, Claire Majewska, Amandine Brunquers, Sumalee Wongpramud, Bénédicte Valet, Philippe Janssens, Monique Dillemans, Laurence Van Nedervelde, Dominique Gallo

**Affiliations:** ^1^Institut Meurice, 1 avenue Emile Gryzon, 1070 Brussels, Belgium; ^2^Yakima Chief-Hopunion LLC, 10 avenue A. Fleming, 1348 Louvain-La-Neuve, Belgium; ^3^Commission Communautaire Française (Cocof), Service des Industries Biochimiques, Belgium; ^4^Commission Communautaire Française (Cocof), Département des Substances Naturelles et de Biochimie, Belgium

## Abstract

Tetrahydro-iso-alpha acids commonly called THIAA or Tetra are modified hop acids extracted from hop (*Humulus lupulus* L.) which are frequently used in brewing industry mainly in order to provide beer bitterness and foam stability. Interestingly, molecular structure of tetrahydro-iso-alpha acids is close to a new type of estrogen receptor alpha (ER*α*) antagonists aimed at disrupting the binding of coactivators containing an LxxLL motif (NR-box). In this work we show that THIAA decreases estradiol-stimulated proliferation of MCF-7 (ER*α*-positive breast cancer cells). Besides, we show that it inhibits ER*α* transcriptional activity. Interestingly, this extract fails to compete with estradiol for ER*α* binding and does not significantly impact the receptor turnover rate in MCF-7 cells, suggesting that it does not act like classical antiestrogens. Hence, we demonstrate that THIAA is able to antagonize ER*α* estradiol-induced recruitment of the LxxLL binding motif.

## 1. Introduction

Estrogen receptor alpha (ER*α*) is a member of the nuclear receptor (NR) family known to act as ligand-dependent transcription factors. Involvement of ER*α* and its natural hormone (17*β*-estradiol; E_2_) in the growth of hormone-dependent breast cancer cells (around two-thirds of breast cancer cases) is established for a long time. Like other members of nuclear receptor family, ER*α* is characterized by five functional domains (i.e., A/B, C, D, E, and F domains). E_2_ binding, in the E-domain, induces several changes in ER*α* conformation leading to the transition of the receptor from an inactive to an active state. In this active state, ER*α* is known to activate the transcription of target genes through DNA binding (C-domain). However, this view should be fleshed out since E_2_-stimulated transcription appears to be a dynamic and multicomponent process [1–3]. Actually, this mechanism involves sequential recruitment and dissociations of a large number of coactivators and corepressors [4]. These proteins play a major role, not only for the onset of the transcription but also in ER*α* behaviors such as translocation, turnover, and crosstalk with other signaling pathways. Hence, in a pharmacological point of view, search for compounds able to disturb ER*α*/coregulators interplay is an attractive approach to modulate the receptor activity (see [5] and references herein).

Today, two therapeutic strategies are proposed for the specific treatment of ER*α*-positive breast cancer. The first one is the direct inhibition of the receptor by using antiestrogens and the second one is the inhibition of E_2_ synthesis by means of aromatase inhibitors. Note that, directly or indirectly, these two strategies target,* in fine*, the hormone binding pocket of ER*α* located in its E-domain. Unfortunately, a proportion of patients are or can become resistant to these drugs [6–8]. Thus, compounds targeting a domain distinct from the ligand binding pocket may offer an alternative therapeutic strategy [5]. In this respect, several compounds aimed at inhibiting coactivator recruitment (i.e., Coactivator Binding Inhibitors (CBIs)) have been developed [9–18]. The key element in this research was the discovery of a consensus sequence at the surface of NR coactivators: the LxxLL motif (L, leucine, and x, any other residue) also called NR-box [19–21]. This motif is found in several NR coactivators such as members of the CBP/p300 and SRC/p160 families. When ER*α* is activated by a ligand, conformational modifications induce the emergence of a hydrophobic groove (i.e., Activation Function-2 (AF-2)) in which leucines can be engulfed. By mimicking this sequence, peptidic and nonpeptidic CBIs are able to competitively inhibit the recruitment of ER*α* coactivators. It is worth noting that nonpeptidic CBIs are uniformly characterized by the presence of hydrophobic lateral chains that mimic leucines of the LxxLL motif [13, 22]. Triazines, pyrimidines, trithianes, cyclohexanes, or pyridyl-pyridines are different examples of scaffolds allowing a suitable orientation of these side chains.

**Figure 1:**
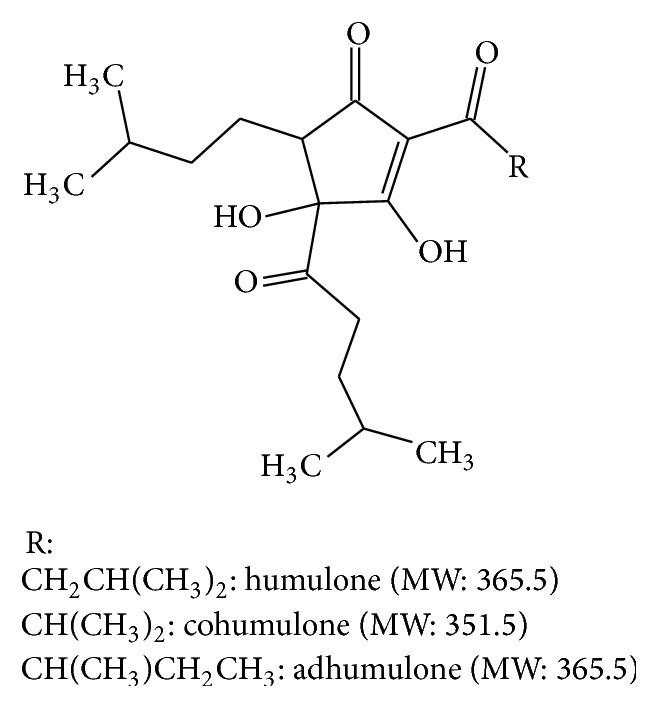
Molecular structure of THIAA.

Interestingly, structure of hop (*Humulus lupulus* L.) *α*-acids and their derivatives are close to nonpeptidic CBIs described here (Figure 1). *α*-acids are a family of structurally similar compounds extracted from the resin of mature hop strobili (see [23] for review). They are mostly used in brewing industry to provide beer bitterness. This family is mainly composed of three molecules, that is, humulone, cohumulone, and adhumulone. Actually, a thermal isomerization (acyloin-type ring contraction) of *α*-acids is required to reach the desired bitterness [24, 25]. This chemical process occurs during wort boiling, before cooling, and fermentation. It generates six compounds:* cis*- and* trans*-isohumulone, isocohumulone, and isoadhumulone. Because these compounds are light-sensitive and generate “stale off-flavor,” stable reduced isomerized *α*-acids are regularly used to avoid this drawback [26]. Three kinds of reduced isomerized *α*-acids are commercially available; they are commonly called Rho, Tetra (hereunder called THIAA, Figure 1), and Hexa referring to the reduction of one, two, or three *π*-bounds, respectively.

In a pharmacological point of view, hop *α*-acids and their derivatives appear to be a promising class of molecules. First, these reduced isomerized *α*-acids molecules are known for their antibacterial properties [27]. It has also been shown that they can restore insulin sensitivity in type II diabetes patients [28]. Furthermore, these compounds are able to inhibit NF-*κ*b, TNF*α*, mTOR, AP-1, and COX-2 [29–31]. Hence, it was shown that they exhibit anti-inflammatory and antiangiogenic properties and induce a proliferation inhibition in various cancer cell lines [32–39]. The present study highlights a new pharmacological property of hop *α*-acid derivatives, that is, THIAA, which impede ER*α* activity in MCF-7 breast cancer cells by a mechanism most probably depending on the recruitment inhibition of coactivators.

## 2. Materials and Methods

### 2.1. Hop Acids

Hop extracts used in this study are commercial preparations obtained from Yakima Chief-Hopunion LLC. Concerning tetrahydro-iso-alpha acids (THIAA), this extract was a clear aqueous solution of the potassium salts of hop-derived THIAA standardized at 9% w/w by HPLC. Before cell treatment, dilutions were made in analytical grade ethanol and percentages given in this work refer to dilutions of commercial preparations (v/v). Concentrations specified in all experiments represent dilutions of this stock solution. Hence, a 0.1% concentration corresponds to a solution of 90 mg/L, which, considering an average molecular weight of 365 g/mol, would represent a concentration of combined components of ca. 0.25 mM. Note that, for cell treatments, final ethanol concentrations do not exceed 0.1% v/v.

### 2.2. Cell Culture

MCF-7, MDA-MB-231, and MVLN cells were propagated at 37°C (5% CO_2_, humid atmosphere) in Earle's based minimal essential medium (EMEM) supplemented with phenol red, 2 mM L-glutamine, 100 U/mL penicillin, 100 *μ*g/mL streptomycin, and 10% heat-inactivated fetal bovine serum (FBS) (all reagents from Invitrogen). This culture condition is referred to hereafter as “serum-complete culture condition". For steroid-free culture condition, experiments were performed in phenol red-free EMEM containing 2 mM L-glutamine, 100 U/mL penicillin, 100 *μ*g/mL streptomycin, and 10% charcoal-stripped FBS.

### 2.3. Proliferation Measurement: Crystal Violet Staining

Cells were seeded in 96-well plates (3000 cells/well). Twenty-four hours after seeding, cells were treated or not (control) for a period of 72 hours. Cell growth was then measured by crystal violet staining as previously described [40]. Briefly, cells were washed with PBS, fixed for 15 minutes in glutaraldehyde (1% in PBS), and stained for 30 minutes with crystal violet (0.1% w/v in distilled water). After removal of dye excess, cell-bound crystal violet was extracted with 1% v/v Triton X-100 and absorbance was measured at 550 nm (Oasis UVM340 spectrophotometer).

### 2.4. Metabolic Activity Measurement: MTT Assay

Cells were seeded in 96-well plates (3000 cells/well). Twenty-four hours after seeding, cells were treated or not (control) for a period of 72 hours. Metabolic activity was then assayed by exposure to 0.03% MTT w/v. After medium removal, produced formazan was dissolved in DMSO (1 hour, room temperature, under agitation) for measurement by spectrophotometry at 550 (Oasis UVM340 spectrophotometer).

### 2.5. Apoptosis Measurement by Flow Cytometry: Annexin V-FITC/Propidium Iodine Labeling

Cells were seeded in 6-well plates (2.5 10^5^ cells/well) and allowed to grow for 24 hours in serum-complete condition. Then, cells were treated or not (control) for a 24-hour period (serum-complete condition). After monolayer trypsinization, cells were labeled with annexin V-FITC and propidium iodine (Dead Cell Apoptosis, Invitrogen) according to manufacturer's instructions. Cytometric analyses were carried out with an Attune acoustic focusing cytometer and data were analyzed using Attune cytometric software V2.1 (Applied Biosystems). Statistical analysis was performed by ANOVA followed by* post hoc* Tukey test (for *p* values <0.05) using Fizz software.

### 2.6. Cell Cycle Measurement by Flow Cytometry

MCF-7 cells seeded in Petri dishes were treated during 72 hours with indicated compounds. After trypsinization and permeabilisation/fixation with 70% ethanol, cells were treated with DNase-free RNase (Invitrogen) and DNA was stained with propidium iodine (Invitrogen) at 10 *μ*g/mL for 15 minutes. After a 2x dilution in PBS, cells were analyzed by flow cytometry. Cytometric analyses were carried out with an Attune acoustic focusing cytometer and data were analyzed using Attune cytometric software V2.1 (Applied Biosystems).

### 2.7. Clonogenicity Study

Cells were seeded in Petri dishes at low concentration (300 cells/well) in steroid-free culture condition. Twenty-four hours after seeding, cells were treated or not (control) with indicated compounds for a 72-hour period. Cells were then fixed and stained with crystal violet as described above. After staining, both number of colonies and number of cells per colony were counted. For the latter, data were subdivided into 4 classes (1-2, 3–6, 7–11, and more than 11 cells) according to a quartile calculation.

### 2.8. ER*α*-Dependent Transcription: Measurement by Luciferase Induction Assay

MVLN cells (MCF-7 stably transfected with pVit-tk-Luc reporter plasmid [41]) were seeded in 6-well plates in steroid-free culture condition and were treated for 24-hour with indicated compounds. Cells were then washed twice with PBS and luciferase activity was measured in cell lysates by luminometry using Luciferase Assay System (Promega) and a GenProbe Leader 50 luminometer. Results were normalized with respect to total protein content measured by BCA method (Pierce).

### 2.9. Western Blot Analyses

MCF-7 cells were seeded in Petri dishes in steroid-free culture condition. After 24 hours of treatment with indicated compounds cells were washed with PBS before lysis in RIPA buffer: TBS containing 1% NP-40, 0.1% SDS, 0.5% sodium deoxycholate, 50 mM NaF, 0.1 mM sodium orthovanadate, 0.6 mM PMSF, 0.3 mM TPCK, and protease/phosphatase inhibitor mix (Fisher Scientific). Lysates were clarified and protein concentration of each sample was determined by BCA assay (Pierce). After addition of LDS Sample 4x buffer (Invitrogen), samples were boiled for 5 minutes and submitted to electrophoresis on a 4–12% SDS-PAGE gel (Invitrogen). Separated proteins were electrotransferred onto Hybond ECL nitrocellulose membrane (GE Healthcare) using a semidry blotting apparatus (BioRad). Nonspecific sites were blocked with 5% nonfat dry milk in TBS containing 0.05% Tween 20 (2 hours, room temperature). Membranes were then incubated overnight at 4°C with primary antibodies (F-10 at 1/750 and MAB1501 at 1/3000 from Santa Cruz Biotechnology and Millipore, resp.). Detection was performed by chemiluminescence, using a peroxidase-coupled secondary antibody (1.5 hours, room temperature) and Western Pico Detection system (both from Pierce). Immunoblots were visualized using a ChemiDoc XRS+ camera and densitometric analyses were performed using ImageLab software (BioRad).

### 2.10. Immunofluorescence

MCF-7 cells were seeded in 8-chamber Lab-Tek slides in steroid-free culture condition and allowed to grow for 24 hours before treatment with or without (control) indicated compounds. After 24 hours of incubation, monolayers were washed twice with PBS, fixed in a 3% paraformaldehyde-PBS solution (20 minutes, room temperature), and permeabilized with a 0.1% Triton X-100-PBS solution (5 minutes, room temperature, under agitation) and nonspecific binding sites were blocked with a 3% BSA-PBS solution (overnight, 4°C). Cells were then successively exposed to the primary antibody (F-10 at 1/100, Santa Cruz Biotechnology), a biotinylated secondary antibody (Pierce), and the DyLight 594-labeled streptavidin (Thermo Scientific). Estrogen receptor was then visualized by fluorescence microscopy (Nikon DXM1200C).

### 2.11. [^3^H]E_2_ Relative Binding Affinity Measurement in MCF-7 Cells

MCF-7 cells were seeded in 24-well plates in steroid-free culture condition and allowed to grow for 48 hours before analysis. After removal of the culture medium, cells were exposed during 40 minutes to radiolabeled E_2_ at 10^−9 ^M (2, 4, 6, 7, 16, 17 ^3^H(N)-estradiol from PerkinElmer) in the presence or absence of increasing concentrations of E_2_ or THIAA in serum-free and phenol red-free EMEM. Nonspecific binding was assessed by addition of a 1000-fold excess of unlabeled hormone. After labeling, cells were washed three times with PBS and cell-associated [^3^H]E_2_ was assessed by liquid scintillation counting (LS6500 scintillation counter, Beckman Coulter) after ethanol extraction.

### 2.12. Assessment of ER*α*-LxxLL Motif Association by TR-FRET

An* in vitro* Time Resolved Fluorescence Resonance Energy Transfer (TR-FRET) assay was carried out to evaluate the association between a recombinant ER*α* containing a GST sequence and a fluorescein-labeled LxxLL peptide (ER*α* LanthaScreen, Life Technologies). This experiment was performed according to manufacturer's instructions. Briefly, the receptor was incubated with or without indicated compounds for 30 minutes on ice. After adjunction of the terbium anti-GST antibody and the labeled peptide, samples were incubated during 1 hour and fluorescence was measured at 488 nm and 518 nm under a 332 nm excitation wavelength using a SpectraMax M5 fluorometer and data were analyzed using the SoftMax Pro software (Molecular Device).

### 2.13. Assessment of ER*α*-LxxLL Motif Association by ELISA

MCF-7 cells were seeded in Petri dishes in steroid-free culture condition and allowed to grow for 48 hours. Then, cells were treated or not (control) during 30 minutes with E_2_ at 10^−9 ^M. Cells were washed twice with PBS and lysed with RIPA buffer. Samples (with or without THIAA or the LxxLL competitor peptide (AnaSpec) at indicated concentrations) were then submitted to an ELISA-based method in which the capture element is an LxxLL peptide (ER*α* Elisa NR peptide, Active Motif) according to manufacturer's instructions. Binding of ER*α* to the plate was measured by colorimetry (measurement at 450 nm, Oasis UVM340 spectrophotometer), after addition of an anti-ER*α* primary antibody and of an HRP-conjugated secondary antibody.

## 3. Results and Discussion

### 3.1. Effect of THIAA on Proliferation and Metabolic Activity of Breast Cancer Cells

**Figure 2:**
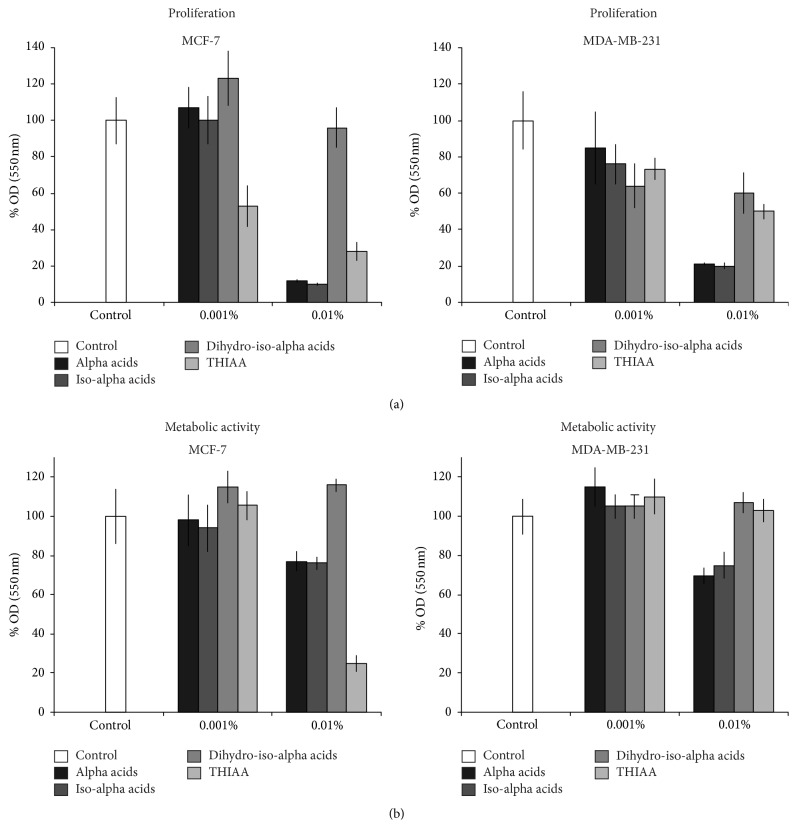
Effect of commercial hop extracts on proliferation (a) and metabolic activity (b) of MCF-7 and MDA-MB-231 cells in serum-complete condition. Cells were grown during 72 h in the absence (control) or presence of indicated hop extracts at 0.001 and 0.01%. Cell proliferation was measured by crystal violet staining and metabolic activity by the MTT method. Measurements were performed in sixplicate. Data refer to the mean value ± SD of a representative experiment performed independently three times.

**Figure 3:**
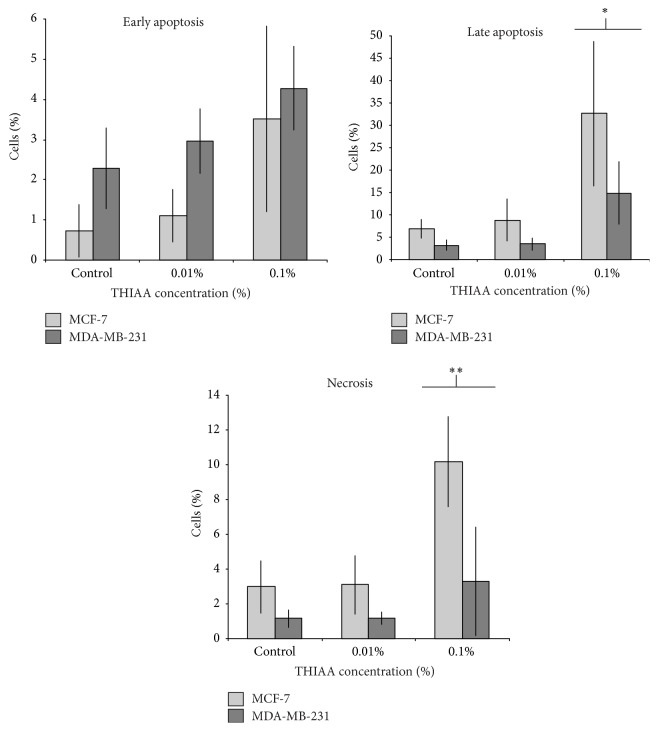
THIAA induced apoptosis in serum-complete condition. MCF-7 and MDA-MB-231 cells were treated (or not: control) during 24 h with indicated concentrations of THIAA. Early and late apoptosis as well as necrosis were assessed by flow cytometry after annexin V-FITC/propidium iodine labeling. Data refer to the mean value ± SD of three independent experiments; ^*∗*^ *p* < 0.2; ^*∗∗*^ *p* < 0.05.

We have first selected a commercial preparation of *α*-acids, or derivatives, able to exhibit a significant effect on MCF-7 cells with a moderate influence on MDA-MB-231 cell line. These two breast cancer cell lines differ from each other by their hormone dependency. While the MCF-7 cells express a high level of ER*α*, the MDA-MB-231 cell line does not. Comparison of results obtained with these cell lines will provide first information about a potential effect on the estrogen receptor. Indeed, these compounds are known to exert cytotoxic actions on diverse cancer cells independently of ER*α* expression [37–39]. Results show that the preparation of tetrahydro-iso-alpha acids (hereafter called THIAA) exerts a higher effect on MCF-7 as compared to MDA-MB-231 cells toward their proliferation and their metabolic activity in serum-complete culture condition (Figure 2). Actually, in this condition (i.e., presence of steroids) THIAA at 0.01% (v/v) induces a proliferation decrease of 72% in MCF-7* versus* 50% in MDA-MB-231 cells. Concerning the metabolic activity, for the same concentration, THIAA induces a decrease of 75% in MCF-7 and failed to affect MDA-MB-231 cells. In order to know how these data are relevant to a potential apoptosis induction, we studied the effect of THIAA on this physiological phenomenon by flow cytometry, for both cell lines (annexin V-FITC/propidium iodine labeling, Figure 3). Results show that, in serum-complete culture condition, THIAA mainly acts by increasing late apoptosis and necrosis (24 hours of treatment). Moreover, like for previous experiments, THIAA appears to be more efficient in MCF-7, as compared to MDA-MB-231 cells.

**Figure 4:**
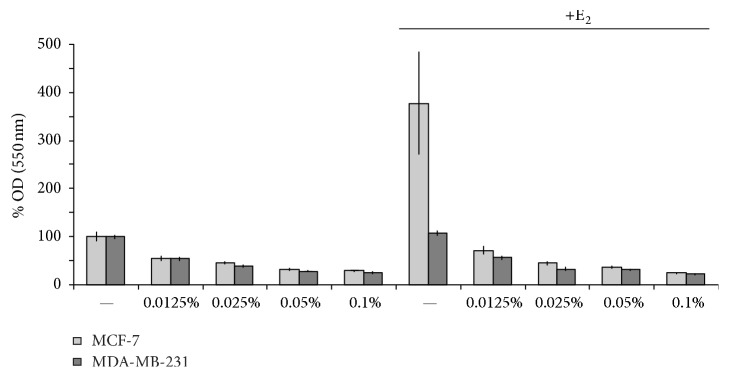
Effect of THIAA on E_2_-stimulated MCF-7 cell proliferation in steroid-free culture medium. MCF-7 cells were grown during 72 h in the absence (control) or presence of E_2_ at 10^−9 ^M with or without THIAA at indicated concentrations. Cell proliferation was measured by crystal violet staining. Data refer to the mean value ± SD of a representative experiment performed independently three times in sixplicate.

**Figure 5:**
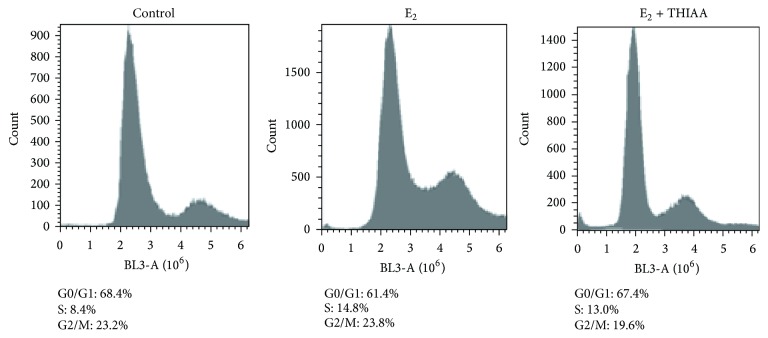
Effect of THIAA on E_2_-stimulated cell cycle progression in steroid-free culture medium. MCF-7 cells were grown during 72 h in the absence (control) or presence of E_2_ at 10^−9 ^M with or without THIAA at 0.01%. Cell cycle phases were analyzed by flow cytometry after propidium iodine labeling. Data refer to a representative experiment performed independently three times.

**Figure 6:**
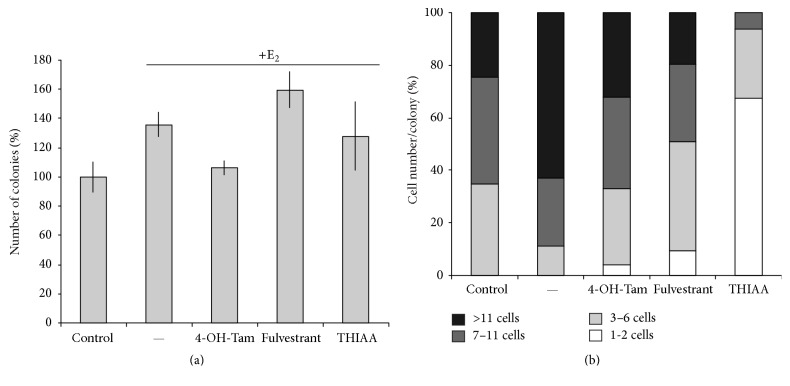
Effect of THIAA on E_2_-stimulated MCF-7 cell clonogenicity in steroid-free culture medium. Cells were seeded in Petri dishes at low concentration. Cells were grown during 72 h in the absence (control) or presence of E_2_ at 10^−9 ^M with or without antiestrogens at 10^−6 ^M or THIAA at 0.01%. Number of colonies (a) and number of cells per colony (b) were counted. For the later, data were subdivided into 4 classes (1-2, 3–6, 7–11, and more than 11 cells) according to a quartile calculation.

Hence, a specific effect of THIAA on the estrogen receptor would be conceivable. Therefore, we evaluated its effect on E_2_-stimulated MCF-7 proliferation (Figure 4, steroid-free culture medium). Interestingly, beside a general cytotoxic effect noticeable on both MDA-MB-231 and unstimulated MCF-7 cells, THIAA appears to exhibit an antiestrogenic effect. Indeed, THIAA completely abrogates the trophic effect of E_2_ on MCF-7 cells. Accordingly, cell cycle analyses by flow cytometry show that THIAA is able to reverse the effect of E_2_ on MCF-7 cell cycle progression (Figure 5). Complementary studies of clonogenicity were carried out in order to confirm these data (Figure 6). Results show that the effect of THIAA is mainly due to a slowdown of the cell cycle. Indeed, while its effect on the number of colonies appears weak, the size of colonies (number of cells/colony) is prominently affected when THIAA is used in cotreatment with E_2_. Of note, when compared to antiestrogens, that is, 4-OH-tamoxifen and fulvestrant at 10^−6 ^M, THIAA at 0.01% appears more effective with regard to the growth arrest of E_2_-stimulated MCF-7 cells.

### 3.2. Effect of THIAA on ER*α* Transcriptional Activity

**Figure 7:**
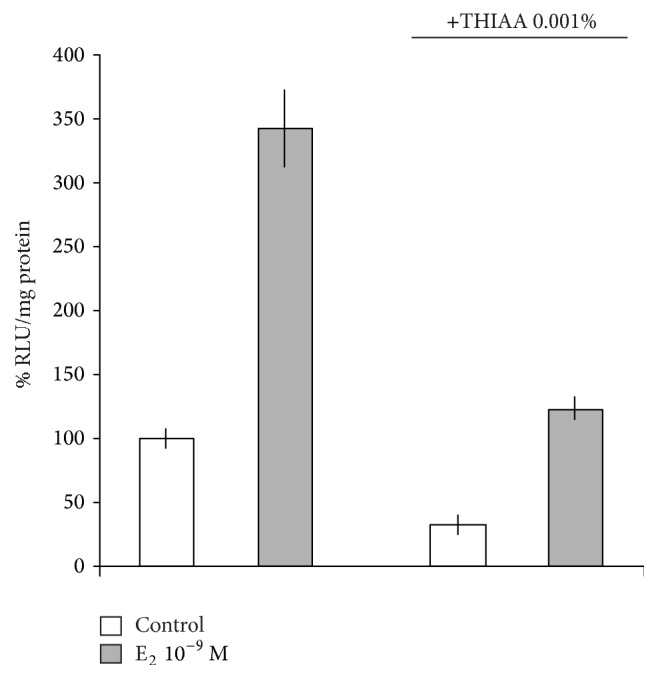
Effect of THIAA on ER*α*-dependent transcription by reporter gene assay in steroid-free culture medium. MVLN cells were incubated for 24 h in the absence (control) or presence of E_2_ at 10^−9 ^M with or without THIAA at 0.001%. Luciferase activity was assayed in cellular extracts by luminometry and emitted light signals were expressed in arbitrary units (relative luciferase units (RLU)) per mg protein. Data refer to the mean value ± SD of an experiment performed in triplicate and are representative of three independent experiments.

**Figure 8:**
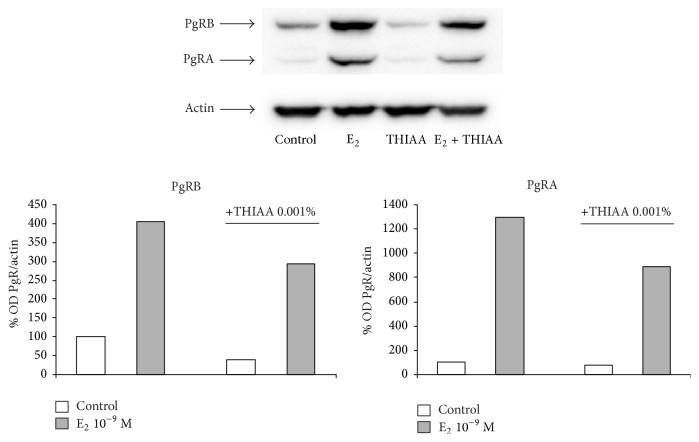
Effect of THIAA on PgR expression in steroid-free culture medium by western blot. MCF-7 cells were incubated for 24 h in the absence (control) or presence of E_2_ at 10^−9 ^M with or without THIAA at 0.001%. Immunoblots and optical density (OD) analyses of PgRA and PgRB refer to an experiment performed independently twice. ODs of both PgR isoforms are normalized by ODs of actin and expressed as percentage of control.

In the context of general (anti-)estrogenic properties, a link between MCF-7 proliferation and ER*α* transcriptional activity is established [3, 42–45]. In this study, experiments performed with MCF-7 cells stably transfected with a Vit-tk-Luciferase reporter gene (MVLN cells [41]; “Vit” refers to the promoter of the vitellogenin A2 of* Xenopus laevis*) exposed to THIAA revealed that the MCF-7 proliferation decrease described here is associated with a reduction of ERE-dependent transactivation (Figure 7). THIAA at 0.001% induces, in this regard, a decrease of both the basal (100% versus 32%) and the E_2_-induced (343% versus 123%) reporter gene activity. Note that, in the same conditions, THIAA failed to reduce Vit-tk-Luciferase reporter gene activity when transiently transfected in the ER*α*-negative Cos-7 cell line (data not shown), supporting the assumption that THIAA acts on ER*α*. In order to confirm these data, we assessed the expression of progesterone receptor (PgR), a standard ER*α*-regulated gene product. Western blot analyses of A and B isoforms of PgR (Figure 8) also show a decrease of ER*α* transcriptional activity even if THIAA efficiency appears lower as compared to the previous experiment. In fact, this decrease is noticeable in both basal (PgRB: 100% versus 39%; PgRA: 100% versus 79%) and E_2_-stimulated (PgRB: 405% versus 293%; PgRA: 1292% versus 887%) conditions.

### 3.3. Effect of THIAA on ER*α* Turnover and ^3^[H]E_2_ Binding Capacity

**Figure 9:**
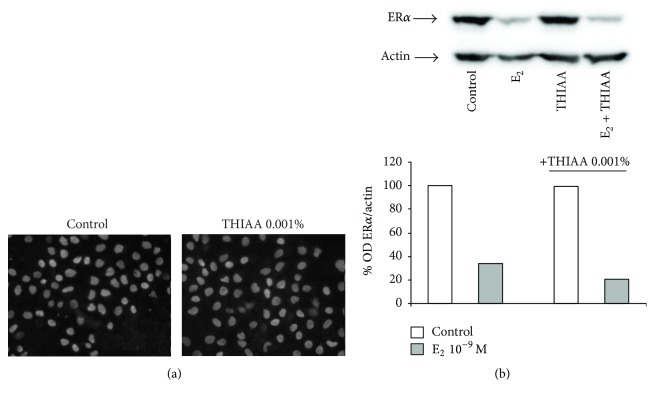
Effect of THIAA on ER*α* protein level in steroid-free culture medium by immunofluorescence (a) and western blot (b). MCF-7 cells were incubated for 24 h in the absence (control) or presence of E_2_ at 10^−9 ^M with or without THIAA at 0.001%. Immunofluorescence, immunoblots, and optical density (OD) analyses of ER*α* refer to an experiment performed independently twice. ODs of ER*α* are normalized by ODs of actin and expressed as percentage of control.

Classical antiestrogens, by inducing several ER*α* conformation changes, impact the receptor turnover rate. While partial antiestrogens, like tamoxifen, upregulate ER*α*, pure antiestrogens, like fulvestrant, induce its rapid proteasomal degradation. In order to evaluate whether the antagonistic properties of THIAA could be attributed to a classical mode of action, ER*α* protein level was assessed by both western blot and immunofluorescence (Figure 9). Results show that after 24 hours of treatment, THIAA does not significantly modulate ER*α* level suggesting that it does not act like conventional antiestrogens. Note that at higher concentrations and duration of treatment, THIAA slightly decreases ER*α* protein level (decrease of 31% after 48 hours of treatment with 0.01% THIAA, data not shown) suggesting that it may display an indirect effect on ER*α* turnover rate.

**Figure 10:**
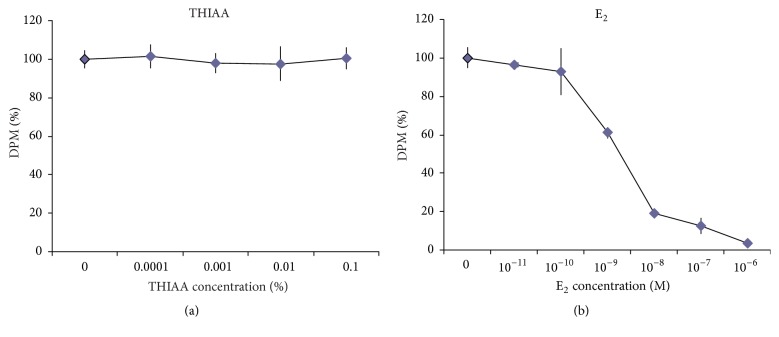
Effect of THIAA on [^3^H]E_2_-ER*α* complexation in MCF-7 cells. MCF-7 cells were incubated during 40 minutes with 10^−9 ^M [^3^H]E_2_ and increasing concentrations of THIAA (a) or E_2_ (b). Whole cell [^3^H]E_2_ binding capacity was assessed by liquid scintillation counting. Data (mean ± SD) refer to a representative experiment performed three times in triplicate.

On the other hand, additional studies show that THIAA has no influence on E_2_-ER*α* association in MCF-7 cells (Figure 10). Indeed, conventional competitive assays indicate that THIAA, up to 0.1%, does not affect [^3^H]E_2_ binding, supporting the fact that it acts through a site distinct from the hormone binding pocket. Note that, even after 6 hours of incubation before binding assay, THIAA at 0.1% does not decrease MCF-7 [^3^H]E_2_ labeling (data not shown, *n* = 3) reflecting its inaction regarding both ER*α* turnover rate and binding capacity.

### 3.4. Effect of THIAA on LxxLL Motif Recruitment

**Figure 11:**
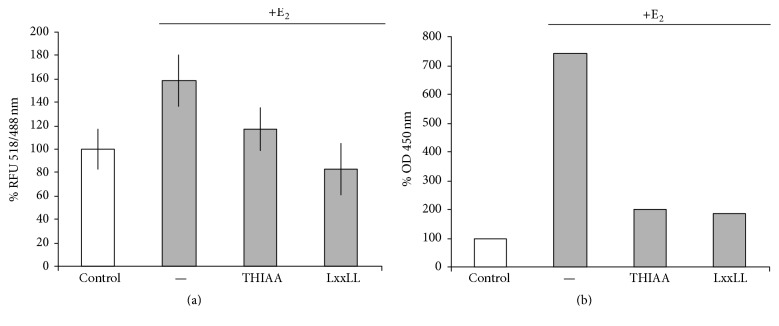
Effect of THIAA on LxxLL motif recruitment assessed by TR-FRET (a) and ELISA (b). For TR-FRET experiments, recombinant ER*α* was incubated in the absence (control) or presence of E_2_ at 10^−6 ^M with or without THIAA at 0.001% or an LxxLL competitor peptide at 10^−4 ^M. Fluorescence emission was measured at 488 nm and 518 nm under a 332 nm excitation wavelength. Data refer to the mean value ± SD of Relative Fluorescence Unit (RLU) ratio at 518 nm and 488 nm of an experiment performed twice in triplicate. For ELISA, cell extracts of MCF-7 treated or not with E_2_ at 10^−9 ^M (30 min) were incubated with or without THIAA at 0.001% or an LxxLL competitor peptide at 10^−4 ^M. Cell extracts where then submitted to this ELISA in which the capture element is an LxxLL peptide. Data refer to an experiment performed three times.

By contrast to classical antiestrogens, CBIs are molecules designed to impede the recruitment of LxxLL-containing coactivators on ER*α* (see [5] for review). To evaluate the potential CBI-like effect of THIAA, an* in vitro* TR-FRET system as well as an* ex cellulo* ELISA-based method were carried out (Figure 11). Note that, in both assays, THIAA failed to produce a significant effect in the absence of E_2_ (data not shown). In the first test that uses a purified recombinant ER*α*, THIAA decreases the ER*α*-E_2_/LxxLL binding like a competitor peptide containing this amino acid sequence (LxxLL peptide). This antagonistic effect appears obvious in the second test that uses E_2_-stimulated MCF-7 cell extracts (untreated cells were used for the control). In this case, treatment of cell extracts with THIAA completely abrogates the effect of E_2_, like the LxxLL competitor peptide. Hence, altogether, these results suggest that THIAA antagonizes the effect of E_2_ through a mechanism that depends on the coactivator binding pocket. In this regard and in view of the THIAA chemical structure, a direct competition with the LxxLL motif is proposed.

## 4. Conclusions

Investigation on natural compounds able to modulate cell signaling remains an attractive approach for the discovery of new drug candidates [46]. Hop (*Humulus lupulus* L.) appears to be a reservoir of such bioactive compounds [47, 48]. Hence, a large number of works has been devoted to the study of hop-derived molecules. Nevertheless, in most cases, these studies highlight biological properties of polyphenols. In this work, we investigated biological effects of *α*-acids and their isomerized and subsequently reduced derivatives. These compounds are usually used in brewing industry to provide beer bitterness. Recently, several studies have shown that *α*-acids derivatives display interesting pharmacological properties. It was indeed shown that these compounds are able to regulate key signaling actors like NF-*κ*b, TNF*α*, mTOR, AP-1, and COX-2 leading to interesting biological effects, like anti-inflammatory and antiangiogenic properties [49].

In this work we have shown that THIAA, an isomerized and reduced *α*-acid preparation, exhibits antiestrogenic properties. Indeed, when given to MCF-7 cells, THIAA abrogates cell proliferation increase induced by E_2_. This trophic effect inhibition is associated with an ER*α* transcriptional activity decrease. The fact that THIAA failed to significantly modulate ER*α* protein turnover and that it is unable to compete with [^3^H]E_2_ for ER*α* binding led us to assume that it does not act like classical antiestrogens through binding into the hormone binding pocket. In this view, THIAA general chemical structure seems to be not suitable for such an interaction. Regarding THIAA chemical structure (Figure 1), a competition with ER*α* coactivators seems more likely. ER*α* coactivator binding site is a hydrophobic groove delimited by residues of helices H3–H5 and H12 of the E-domain in which leucines of the LxxLL motif can be engulfed [21]. One may consider that hydrophobic side chains of THIAA, by mimicking leucine residues, can interact with this binding site and, thereby, impede the recruitment of coactivators. This hypothesis is corroborated by* in vitro* and* ex cellulo* experimental data demonstrating that THIAA inhibits E_2_-induced ER*α* LxxLL motif recruitment.

Several compounds, called CBIs, have been synthesized to impede ER*α*/coactivators association. CBIs mainly act by producing a competitive inhibition in a site distinct from the ligand binding pocket. This approach is considered to be a valuable therapeutic strategy, especially when pharmacological ligands appear devoid of efficacy [5]. In this view, a high proportion of patients is, or can become, resistant to conventional antihormone therapies. Hence, new therapeutic modalities are required to overcome ER*α*-positive breast tumors with* de novo* or acquired mechanisms of resistance. By highlighting a CBI-like effect of THIAA, this work could provide basis for the future development of new potent ER*α* antagonists. First-line approaches for the design and hemisynthesis of such compounds will be the identification of the *α*-acid derivative displaying the highest affinity for the LxxLL binding groove as well as docking methodologies.
